# Scoliosis and sagittal balance in Parkinson’s disease: analysis of correlations

**DOI:** 10.1186/1748-7161-8-S2-O7

**Published:** 2013-09-18

**Authors:** Luciano Bissolotti, Massimiliano Gobbo, Fabio Zaina, Monia Lusini, Sabina Donzelli, Stefano Negrini

**Affiliations:** 1 Rehabilitation Service, Casa di Cura Domus Salutis, Brescia, Italy

## Background

Information concerning scoliosis in Parkinson’s disease (PD) and its correlations with sagittal balance (SB) is sparse.

## Purpose

The aim of this study was to describe the prevalence of scoliosis in PD patients and the existing correlations with SB in relation to the spinopelvic morphology.

## Methods

A total of 48 consecutive PD patients were included: 36 males, 12 females; 70.8±7.6 years; 6.4±4.1 years of disease (YOD); Hoehn Yahr (HY) 2.7±1.2. The clinical assessment included HY score, Pain NRS 0-10 and trunk rotation in bending (ATR). Lumbar lordosis (LL), thoracic kyphosis (TK), scoliosis curves (SC), spinosacral angle (SSA), spinopelvic angle (SPA), pelvic incidence (PI), sacral slope (SS) and pelvic tilt (PT) were radiographically assessed. Patients have been compared according to the presence of SC >10° (PD_ts_) Cobb or the absence of SC (PD_ns_).

## Results

Among the study subjects, 47.9% presented a SC larger than 10°, 84% of the patients in PD_ts_ presented a thoracolumbar curve, 10% a thoracic curve and 6% a lumbar curve. The cohort did not present differences with PD_ns_ about age (71.8±6.0 vs. 69.8±8.8yrs) and YOD (6.1±4.1 vs. 6.6±4.1 years). No differences have been detected for HY score (2.7±1.2 vs. 2.6±1.6) and NRS (29.6±22.6 vs. 19.4±28.1). ATR was higher in PD_ts_ (5.6±4.9 vs. 1.3±1.9, p<0.01). TK (46.4±16.1 vs. 46.9±12.1°), LL (46.3±26.9 vs. 49.3±13.9°), SSA (104.8±24.7 vs. 118.6±12.9°) and SPA (152.4±20.3 vs. 153.4±12.5°) were not different (p>0.05). PI (57.8±11.1 vs. 53.9±13.1°) and PT (23.6±13.7 vs. 17.6±8.6°) were slightly but not statistically different, while SS was not (35.3±12.1 vs. 36.0±8.5°).

## Conclusions and discussion

The prevalence of scoliosis in PD was higher than previously described by other authors, with the thoracolumbar spine mostly affected. SB was not different between two groups while, in PD_ts_, spinopelvic parameters presented the tendency to have a larger PI and PT.
